# Orthodontic Treatment of Bilateral Impacted Mandibular Canines and a Mupparapu Type 2 Transmigration

**DOI:** 10.1155/2019/7638959

**Published:** 2019-09-11

**Authors:** José Antonio Vera-Guerra, José Rubén Herrera-Atoche, Gabriel Eduardo Colomé-Ruiz

**Affiliations:** ^1^Department of Orthodontics, School of Dentistry, Universidad Autónoma de Nuevo León, Nuevo León, Mexico; ^2^Department of Orthodontics, School of Dentistry, Universidad Autónoma de Yucatán, Yucatán, Mexico

## Abstract

Dental transmigration is a rare condition that mainly affects the mandibular canines. Since the tooth involved is usually impacted and its crown has crossed the midline towards the opposite side, the treatment options frequently are surgical removal or radiographic follow-up, and, in some cases, orthodontic traction is possible. In 2002, Mupparapu presented a classification for lower canines in transmigration according to their position within the mandible. This paper is aimed at describing the orthodontic treatment of a female patient with two impacted mandibular canines, one of them in a Mupparapu type 2 transmigration position (horizontal impaction position near the lower mandibular border and below the incisors' root apices). Additionally, the paper discusses the biomechanical orthodontic design and the alternative treatment options for these complex cases.

## 1. Introduction

Mandibular canine impaction is one of the most complex eruption dental anomalies to treat, and it occurs mainly when the crown of the affected tooth has crossed the midline and become a transmigration case [1]. According to the literature, the mandibular canines get impacted less frequently than the maxillary ones [2–5]; their prevalence is said to be between 0.92% and 5.1%, and they are usually accompanied by odontomas, cysts, and lateral incisor anomalies, which is the reason why those are associated with etiological factors [6]. Likewise, many studies in different populations report that bilateral impaction is less frequent than unilateral impaction [7–10].

Even though lower canine impaction is less common than upper canine impaction, the first type is much more affected by transmigration [5, 8] (approximately 40.4% of mandibular impacted canines) [11]. On the other hand, it is more frequent in women [1, 5, 7, 10, 12, 13].

According to Mupparapu in 2002, mandibular canines in transmigration might be classified into 5 different types: (1) in a mesioangular position across the lower midline, (2) when the affected canine is in a horizontal impaction position near the lower mandibular border and below the incisor's root apices, (3) when the canine erupts either mesial or distal to the canine of the opposite side, (4) in a horizontal impaction position near the mandibular lower border and below the molars or premolars' root apices of the opposite side, and (5) in a vertical position on the lower midline but with its long axis crossing it [1].

A 2017 systematic review regarding impacted canines found that the treatment options for this condition differ if the affected tooth is just impacted or if it is impacted and in a transmigration condition. For the first, surgical removal and orthodontic traction used to be the most common options; meanwhile, for the latter, surgical removal and a radiographic follow-up tended to be the most frequent [6].

In any case, early diagnosis [11] and treatment [14] are recommended for eruption anomalies such as these, mainly due to the proven incidence of root resorption of the adjacent teeth [11].

This paper is aimed at describing the orthodontic treatment of a female patient with bilateral impaction of mandibular canines (one of them in Mupparapu type 2 transmigration) and at discussing the importance of the biomechanical design for successful treatment of these dental anomalies.

## 2. Case Report

### 2.1. Diagnosis and Etiology

**Figure 1:**
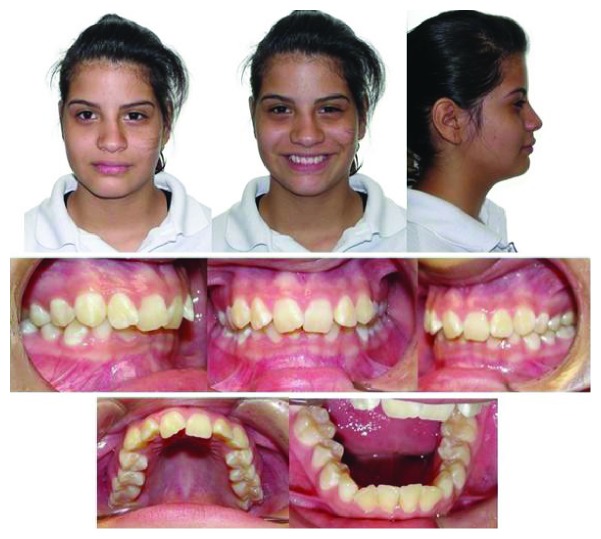
Pretreatment facial and intraoral photographs.

**Figure 2:**
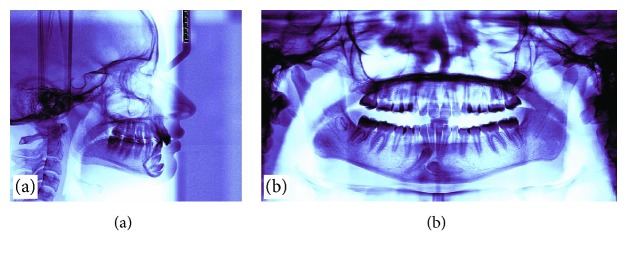
(a) Pretreatment lateral cephalogram. (b) Pretreatment panoramic radiograph.

A 14-year-old female patient was presented for orthodontic evaluation; her chief complaint was the absence of both lower canines. During the clinical assessment, it was noted that the deciduous lower canines were still present and a class I malocclusion with crowding, deep bite, and a straight profile was diagnosed (Figure 1). Dental casts, intraoral and extraoral photographs, and panoramic and lateral cephalogram X-rays were taken. On the panoramic X-ray, it was determined that both mandibular canines were impacted and the left one was in a Mupparapu type 2 transmigration position. Another critical issue was that the crown of the right canine was above the contralateral one. In the lateral cephalogram X-ray, it is possible to see that both canines were towards the vestibular side; however, the right one was closer to the incisors' roots (Figure 2). According to the Ricketts cephalometric analysis (Table 1), it was diagnosed that the lower incisors were retroclined, possibly because of the absence of the canines.

### 2.2. Treatment Objectives

The objectives of the treatment were (a) to open the space for the impacted and transmigrated canines, (b) to traction both canines to a functional position within the dental arch, (c) to improve the buccolingual inclination of the lower incisors, and (d) to correct the deep bite.

### 2.3. Treatment Alternatives

After the analysis of the studies, the patient was presented with the following treatment options: (a) surgical exposure of the canines and orthodontic traction and (b) surgical removal and an orthodontic opening of the spaces for future restoration. The orthodontist explained to the patient's parents that the first option presented a risk of damaging the incisors roots, so the biomechanical design had to consider that. Also, the time of treatment could be longer than when choosing the second option. On the other hand, given her young age, the second option also had a disadvantage in that the patient would wear the restorations for a long time. Moreover, the increment in cost that the restorations would represent should be considered. In light of this information, the patient chose the first option of treatment.

### 2.4. Treatment Progress

**Figure 3:**
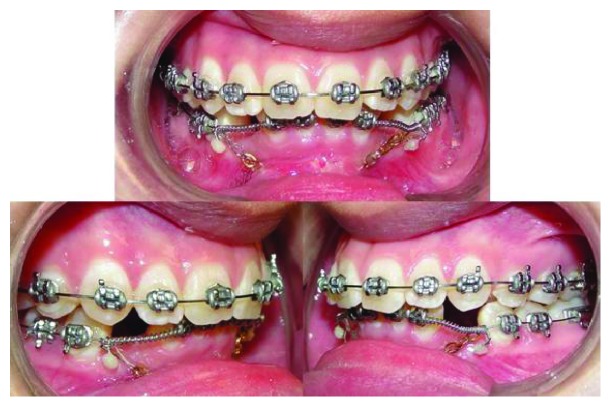
Intraoral photographs. Both golden chains are tied up to the distal end of their respective open coil to start the traction of the impacted canines.

Full fixed 0.018-inch metal Roth brackets were placed and bonded in both arches. The leveling and aligning phase was carried out in both arches using the next sequence of nickel-titanium wires: 0.012, 0.014, and 0.016. This phase lasted six months. Once the patient reached the 0.016 stainless steel (SS) wires, the canines were surgically exposed and two golden chains were cemented for traction. At the same time, to open the space for the canines, a couple of open coil springs were placed and the golden chain was ligated to the distal end of each one to initiate the traction with an up-and-distal vector (Figure 3).

**Figure 4:**
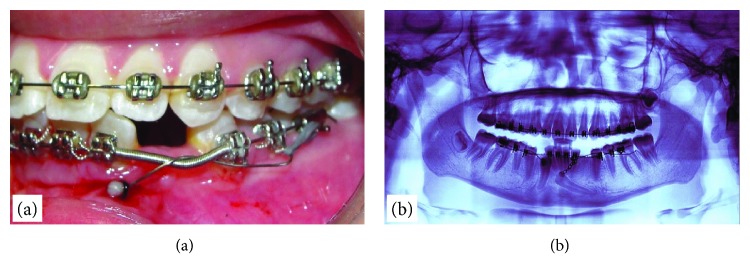
(a) An intraoral photograph of the quadrant 3 accessorial wire tied up to the golden chain. (b) Progress panoramic radiograph.

Posteriorly, three wires were used, a 0.016 SS wire, which played an anchorage role, and two 0.014 nickel-titanium accessorial wires (one per side) to apply the traction force on the impacted teeth. As can be seen in Figure 4, the accessorial wires were tied around the SS wire and their mesial ends were introduced through the link closest to their respective chains (left and right). To prevent the mesial end of each accessorial wire from coming out, they were covered with a composite; meanwhile, their distal ends were bent to hold a power chain that would be tied to their respective first molar tube. With this system, the canines kept receiving a force with an up-and-distal vector. Given the caliber and the alloy of the accessorial wires, the forces were small and the moments were reduced. This scheme translated into having better control of the traction movement.

**Figure 5:**
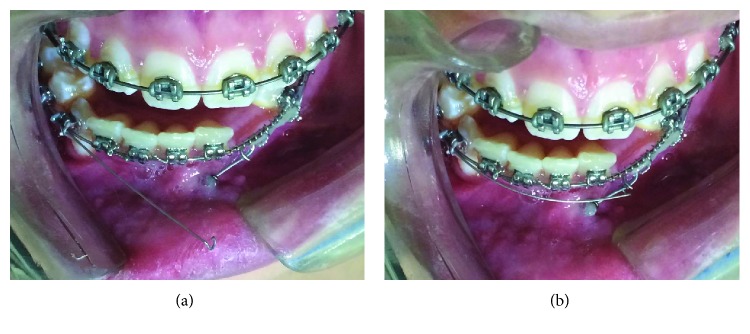
Intraoral photographs showing the accessory wire used to move the crown of the left canine away from the incisors' roots: (a) in its passive manner; (b) once it has been activated.

After seven months, the right canine made erupted and a bracket was fixed on it to be included in the arch. In addition, a new accessorial wire was made to move the crown of the left canine away from the incisors' roots (0.018 nickel-titanium); this wire was tied at the right side (quadrant 4), and it would apply a force towards the vestibule. At the same time, the accessorial wire of quadrant 3 was changed to a 0.016 SS wire, and its mesial end was passed through the first link of the golden chain exposed in the mouth; to keep it from coming out, it was also covered with a composite. The distal end was bent to tie up a power chain that would exert a force with a distal vector (Figure 5). The new wire of quadrant 4 was tied up near the distal end of the sectional wire of quadrant 3; this way, the first would have the freedom to slide through the latter while tractioning the transmigrated canine towards the vestibular end.

**Figure 6:**
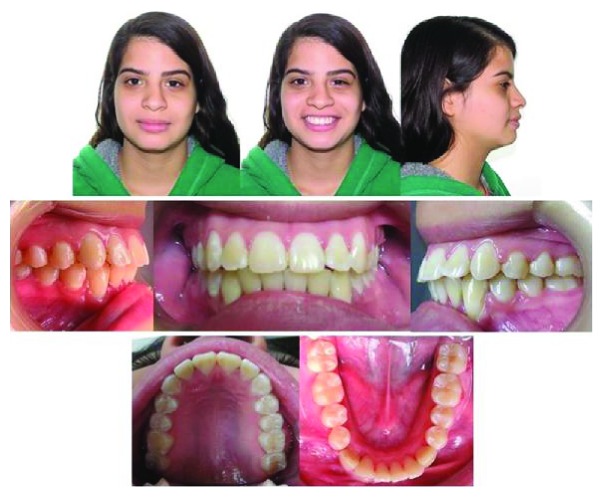
Posttreatment facial and intraoral photographs.

Finally, after the eruption of the left canine, a bracket was fixed on it so it could be included in the dental arch. The next steps were to level the arch again, close the remaining spaces, and do the final detailing. After three years of treatment, the brackets were removed and a fixed retainer was placed in the lower arch while a Hawley retainer was given for the upper section with the indication of full-time use.  Figure 6 shows the case's final photographs.

## 3. Results

**Figure 7:**
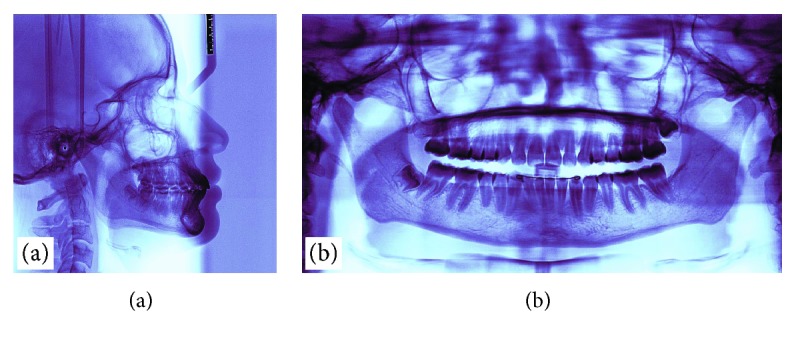
(a) Final lateral cephalogram. (b) Final panoramic radiograph.

**Figure 8:**
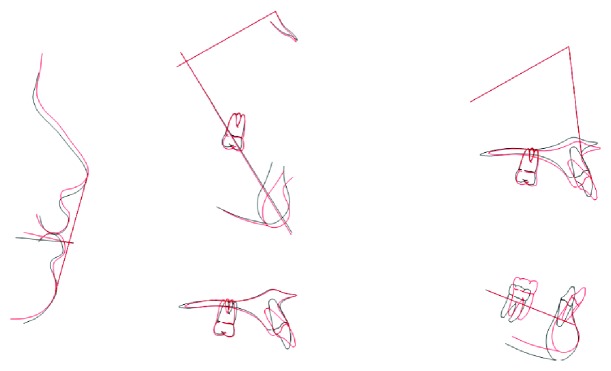
Ricketts superimpositions.

Both canines were successfully brought to a functional position within the dental arch and with a healthy periodontium. At the same time, the opening of the space for the canines helped to correct the buccolingual inclination of the lower incisors and the deep bite. The upper incisors suffered a slight proinclination; however, the patient's profile was kept inside the norm value (Table 1, Figures 7 and 8). Through orthodontic treatment, the crowding, irregularity, and rotations were resolved and, in the end, proper occlusion was achieved. Regarding the aesthetic aspect, a nice smile arc without buccal corridors or an excessive gingival display was obtained; in addition, the straight profile was maintained.

## 4. Discussion

The orthodontic treatment accomplished the goals of bringing both mandibular canines into the dental arch and correcting the crowding and the deep bite, thus achieving a proper occlusion. Moreover, the smile was improved, and at the same time, the treatment did not affect the original profile.

Scientific literature points out that the treatment of transmigrated mandibular canines usually involves a surgical extraction or a radiographic follow-up [6, 7, 11, 15]. Some authors indicate that the orthodontic traction treatment might be possible when the canine is in an angular position, which could be the reason why the Mupparapu type 1 cases are most commonly treated with this modality [16–19]. There are also reports of type 5 cases (vertical position on the lower midline but with its long axis crossing it) treated with orthodontic traction. In these cases, the canine is either tractioned or allowed to erupt, and then, it is shaped with restorative treatment to look like an incisor. Sometimes, this is done to replace some tooth that is missing or damaged by the transmigrated canine [20, 21].

The case described in this paper is the orthodontic traction of a Mupparapu type 2 transmigrated canine, which is unusual since those cases are frequently surgically removed [21, 22], left under radiographic follow-up [7, 18], or even surgically transplanted [23]. In the literature review, there was only one other report that found a Mupparapu type 2 canine that was orthodontically treated. In that case, the authors expressed their concern about damaging the lateral incisor's root during the treatment, so they instead chose to distalize it and then move the transmigrated canine towards its position. After this, both the lateral incisor and the canine were reshaped to resemble the teeth they were replacing [24]. Following other authors' recommendations in the case here described, the orthodontic traction was done with light force [14, 16] and with a biomechanical design that allowed for proper control of the vectors and the amount of force applied to the teeth involved [16]. In this regard, the use of the accessorial wires was critical to control the vectors in order to avoid damaging the adjacent structures.

As a further problem, the patient had the contralateral canine impacted, with its crown above the tooth in transmigration. Likewise, Kuftinec et al. in 1995 described a bilateral transmigration case where both canines were in a similar pattern to the case presented here; given its difficulty, the authors chose to extract both teeth [25]. On the other hand, as can be seen in the case discussed in this paper, it might be advantageous to traction the canine that is closest to the surface to liberate the path for the other.

Finally, in 1994, Wertz suggested that if the canine cusp has gone beyond the apex of the lateral incisor of the opposite side, it might be mechanically impossible to traction it [26]; it is interesting that, despite the fact that not all Mupparapu type 2 transmigrated canines reach this boundary determined by Wertz, most of them are surgically removed or left under radiographic follow-up, a fact that emphasizes the difficulty in treating these cases.

## 5. Conclusions

In conclusion, the traction of impacted mandibular canines is always a challenge for the orthodontist, especially when they are in a transmigrated position. The biomechanical design is critical for the success in these cases; it is recommended to use light forces for better control of vectors given that there is a high risk of damaging the adjacent tooth roots, even more so in Mupparapu type 2 cases.

## Figures and Tables

**Table 1:** Ricketts cephalometric measurements.

	Initial	Final	Norm	Std dev
Dental relationships				
Molar relation (mm)	−0.1	−2.3	−3.0	1.0
Overjet (mm)	5.8	2.7	2.5	2.5
Overbite (mm)	4.8	−0.1	2.5	2.0
Mand incisor extrusion (mm)	2.4	−0.1	1.2	2.0
Interincisal angle (U1-L1) (°)	141.3	111.8	130.0	6.0
Skeletal/dental				
U-incisor protrusion (U1-APo) (mm)	3.3	6.3	3.5	2.3
L1 protrusion (L1-APo) (mm)	−2.0	3.6	1.0	2.3
U-incisor inclination (U1-APo) (°)	17.3	32.8	28.0	4.0
L1 to APo (°)	21.3	35.4	22.0	4.0
Occ plane to FH (°)	15.1	10.4	7.5	5.0
U6-PT vertical (mm)	9.5	16.5	17.0	3.0
Maxillo-mandibular relationships				
Convexity (A-NPo) (mm)	2.1	2.4	0.9	2.0
Mandibular arc (°)	36.7	43.8	29.7	4.0
Craniofacial relation				
FMA (MP-FH) (°)	30.2	20.4	24.2	4.5
Maxillary depth (FH-NA) (°)	87.0	97.1	90.0	3.0
Facial axis-Ricketts (NaBa-PtGn) (°)	92.4	92.0	90.0	3.5
Facial angle (FH-NPo) (°)	84.5	94.5	88.3	3.0
Facial taper (°)	65.3	65.1	68.0	3.5
Deep skeletal structure				
Porion location (mm)	−36.0	−37.6	−38.6	2.2
Cranial deflection (°)	19.6	29.8	27.3	3.0
Ramus position (°)	64.9	71.2	76.0	3.0
Lower face height (ANS-Xi-Pm) (°)	34.8	36.1	45.0	4.0
Esthetic				
Lower lip to *E*-plane (mm)	−1.7	−1.1	−2.0	2.0
